# Organizational factors associated with health worker protection during the COVID-19 pandemic in four provinces of South Africa

**DOI:** 10.1186/s12913-021-07077-w

**Published:** 2021-10-11

**Authors:** Muzimkhulu Zungu, Kuku Voyi, Nosimilo Mlangeni, Saiendhra Vasudevan Moodley, Jonathan Ramodike, Nico Claassen, Elizabeth Wilcox, Nkululeko Thunzi, Annalee Yassi, Jerry Spiegel, Molebogeng Malotle

**Affiliations:** 1 National Institute for Occupational Health, a division of the National Health Laboratory Service, Johannesburg, 2000 South Africa; 2 School of Health Systems and Public Health, University of Pretoria, Pretoria, South Africa; 3 School of Medical and Health Science, Edith Cowan University, Perth, WA Australia; 4 School of Population and Public Health, University of British Columbia, Vancouver, British Columbia V6T 1Z3 Canada; 5 Department of Community Health, Sefako Makgatho Health Sciences University, Ga-Rankuwa, 0208 South Africa

**Keywords:** SARS-CoV-2, Occupational safety and health systems, HealthWISE

## Abstract

**Background:**

Health workers, in short supply in many low-and-middle-income countries, are at increased risk of SARS-CoV-2 infection. This study aimed to assess how South Africa, prepared to protect its health workers from SARS-CoV-2 infection.

**Methods:**

This was a cross-sectional study design applying participatory action research in four provinces of South Africa. A semi-structured questionnaire and a qualitative observational HealthWISE walkthrough risk assessment was carried out to collect data on occupational safety and health (OSH) systems in 45 hospitals across four provinces to identify factors associated with health worker protection. Adapting the International Labour Organization (ILO) and World Health Organization (WHO) HealthWISE tool, we compiled compliance scores through walkthrough surveys. We used logistic regression to analyze the relationship between readiness indicators and the actual implementation of protective measures.

**Results:**

We found that health facilities in all four provinces had SARS-CoV-2 plans for the general population but no comprehensive OHS plan for health workers. Provincial Departments of Health (PDoH) varied in how they were organized to respond: Provinces A and D had an OSH SARS-CoV-2 provincial coordinating team and a dedicated budget for occupational health; Province A had an occupational health doctor and nurse; while Province B had an occupational health nurse; Province A and D PDoHs had functional OSH committees; and Province D had conducted some health risk assessments specific to SARS-CoV-2. However, none of the assessed health facilities had an acceptable HealthWISE compliance score (≥ 75%) due to poor ventilation and inadequate administrative control measures. While the supply of personal protective equipment was adequate, it was often not worn properly. Our study found that having an OSH SARS-CoV-2 policy was significantly associated with higher personal protective equipment and ventilation scores. In addition, our analysis showed that hospitals with higher compliance

scores had significantly lower infection rates (IRR 0.98; 95% CI: 0.97, 0.98).

**Conclusions:**

Despite some initial preparedness, greater effort to protect health workers is still warranted. Low-and-middle-income countries may need to pay more attention to OSH systems and consider using tools, such as ILO/WHO HealthWISE tool, to protect health workers’ health.

**Supplementary Information:**

The online version contains supplementary material available at 10.1186/s12913-021-07077-w.

## Background

The World Health Organization (WHO) declared severe acute respiratory syndrome coronavirus 2 (SARS-CoV-2) a Public Health Emergency of International Concern and subsequently a Global Pandemic on January 30 and March 11, 2020, respectively [1, 2]. As of April 30, 2021, globally, there were 150,110,310 cases and 3,158,792 deaths from coronavirus disease 2019 (COVID-19) [3]. South Africa, a low-and-middle-income country (LMIC) in Sub-Saharan Africa, had 1,581,210 cases and 54,350 deaths in the general population [4]. The National Institute for Occupational Health (NIOH) in South Africa reported that out of 231,552 COVID-19 hospital admissions recorded up to April 10, 2021, 6877 (2.9%) were health workers [5].

Lan et al., [6] reported that health workers were at high risk along with other occupational groups and noted that their COVID-19 infection rate occurrence time-lagged as a reflection of heightened infection prevention and control (IPC) measures in health settings [6]. Health workers were also among the highest risk occupations for exposure to the previous severe acute respiratory syndrome (SARS) pandemic [7]. They were further identified as a high-risk occupational group in Asian countries during both the SARS and the Middle East respiratory syndrome (MERS) coronavirus outbreaks [8]. Clinicians and those performing aerosol-generating procedures in particular account for a significant proportion of SARS-CoV-2 infection and may experience exceptionally high infection incidence following unprotected exposure [9].

There have been conflicting reports on the risk of SARS-CoV-2 infection in health workers. Canova et al., [10] reported a low risk of infection of health workers during routine short clinical examination and short physical contact [10]. Folgueira et al., [11] reported ‘no statistically significant difference in the proportion of SARS-CoV-2 positive polymerase chain reaction (PCR) detection between health workers from high-risk areas involved in close contact with COVID-19 patients in comparison with clerical, administrative, or laboratory personnel without direct contact with patients’ [11]. However, early reports from China suggested that health workers were at an increased risk of SARS-CoV-2 [12, 13]; and the SARS-CoV-2 risk was dependent on performing high-risk work associated with respiratory aerosol production, long work hours and suboptimal hand hygiene [14].

An occupational safety and health (OSH) system is a sub-system of a country’s overarching health systems; South Africa has a unified health system under the National Department of Health with service delivery in public and private sectors [15]. Service delivery in the public sector is the responsibility of nine provincial departments of health (PDoHs). According to the WHO, a health system comprises six building blocks: leadership/governance (health policy, accountability and transparency, and coordination); financing; health workforce; medical products and technology; information, and service delivery [16, 17]. Since standardized health system components model is not disease specific, jurisdictions such as South Africa can readily utilize it in assessing the SARS-CoV-2 readiness of OSH systems at a PDoH level [17]. In South Africa, health and safety committees (HSC) are mandatory under the *Occupational Health and Safety Act*. However, the effectiveness of these bodies is varied and a matter for continued strengthening [18].

Health workers, themselves an essential component in the six health system building blocks described by WHO [16], continue to be a scarce vital resource in the fight against SARS-CoV-2, especially in LMICs. The WHO estimates a projected shortfall of about 18 million health workers by 2030; this shortfall is even more critical in South East Asia and the African regions [19, 20]. There are international calls for LMICs, especially in South East Asia and the African region, to put in place OSH systems and SARS-CoV-2 IPC measures to protect health workers and guard against the collapse of health services secondary to COVID-19. Additionally, protecting health workers is a matter of social justice for this occupational group, irrespective of the legal obligation to provide a safe work environment or the need for health workers to provide care for others.

South Africa has been preparing for SARS-CoV-2 since early February 2020, mainly to protect its health workers before the upsurge of cases in the southern hemisphere. These interventions rely on a strong OSH system and possible utilization of affordable and cost-effective OSH tools, such as the joint International Labour Organization (ILO) and WHO’s Work Improvement in Health Services (HealthWISE) [21]. Two of HealthWISE’s four principles are building on local practices and resources and promoting learning-by-doing.

HealthWISE is a participatory tool for identifying potential hazards (such as SARS-CoV-2) and implement relevant control measures in LMICs. In the COVID-19 pandemic context, HealthWISE encourages managers and staff to work together to improve SARS-CoV-2 IPC and OSH interventions [21]. South Africa participated in the pilot of the HealthWISE tool [22]. Its effectiveness in improving OSH in health care facilities has been evaluated previously in South Africa [22], but not in the specific context of protecting health workers against SARS-CoV-2.

We aimed to explore the extent to which South Africa has been abiding by its legal and social responsibility to protect health workers, a potentially vulnerable workforce by 1) assessing the readiness of OSH systems in place to protect health workers from SARS-CoV-2 in four PDoHs using the WHO’s health system framework; 2) conducting a health facility assessment for the protection of health workers against SARS-CoV-2 infection based on principles of the HealthWISE tool; and 3) ascertaining the relationship between, on the one hand, the existence of policies and/or easy-to-use tools such as HealthWISE, and, on the other hand, the implementation of concrete OSH programmes and/or protective measures at the time (April – June) that WHO declared a global pandemic.

## Methods

### Study design

We used a cross-sectional study design applying participatory action research (PAR) principles to enable action and empower active participation in strengthening OSH [23]. Our study design and methods were selected to enable interaction between the researchers and participants at all stages, including data collection. The PAR design was chosen for its blurring of the distinction between ‘researcher’ and ‘researched’, since all involved in this project participated in all stages of generating knowledge and its implementation, allowing both learning and putting the knowledge generated about OHS COVID-19 interventions into action to protect health workers [24, 25]. We also provided advisory and collective steps for corrective measures where necessary to prevent or halt the spread of SARS-CoV-2 among health workers using an adopted HealthWISE tool. We ensured that responsibility and decisions necessary for action were shared and acted upon between the research team, managers and health workers. We conducted the research in collaboration with the participants as active partners to promote sustainability in line with both HealthWISE and PAR principles [21, 23]. This research was part of a larger study, with four different components, including a COVID-19 knowledge attitudes and practice survey, in which some of our methods were described [26].

### Study setting and population

The study setting was public sector facilities in Gauteng, Limpopo, Mpumalanga, and North-West Provinces of South Africa. These facilities are government-run (by provincial departments of health) and provide services to mainly the uninsured population. The facilities included in the study comprised hospitals at different levels of care (district, regional, tertiary, central, and specialized psychiatric) and community health centres across urban and rural settings. We refer to participating provinces as Province A - D (assigned randomly) in the remainder of the paper to maintain confidentiality and a culture of ‘no blame,’ following the principles of the HealthWISE tool.

The study population included PDoHs OSH managers (completed the study questionnaires), and OSH professionals and health workers (conducted an inventory of the OSH state using the HealthWISE tool but did not provide any individual level information for the research). Health workers included managers, IPC professionals and other frontline staff from all participating PDoHs.

### Sampling

We premised our study sampling on the operational needs of the participating PDoHs and South Africa’s newly formed COVID-19 OSH work steam (committee) under the National Department of Health. It was under this committee that the four participating PDoHs were allocated to the researchers in this study for OSH technical support. The researchers working with the PDoH officials then conveniently selected a total of 45 health facilities based on perceived operational risk of SARS-CoV-2, to include both urban and rural health facilities. Purposive sampling was utilized to select PDoHs respondents and health workers (to conduct the OSH inventory based on HealthWISE tool) from the participating health facilities in consultation with hospital management. Respondents were selected because of their likely knowledge of the systems in question and with the intent of ensuring that a variety of views would be represented. Respondents therefore included OSH and environmental health professionals, unit managers, and trade union representatives.

### Measurement tools and data collection

We developed an interviewer-driven semi-structured questionnaire based on the WHO building blocks of a health system [16], the ILO Convention 161 [27] and a multi-country OHS survey by Rantanen et al. [28], with closed-ended questions. Two occupational hygienists and two occupational medicine doctors tested the feasibility of the questionnaire. Lastly, we collected data from four PDoHs OSH managers on the SARS-CoV-2 readiness of the provincial governments.

We also used an adapted HealthWISE tool [21]—incorporating some elements of the CDC guidelines for TB IPC [29]—to conduct a qualitative observational HealthWISE walkthrough risk assessment of the health facilities. During the walkthrough assessment, the researchers and facility health workers assessed distinct work areas: i) the main passenger and vehicle entrance (staffed by security personnel); ii) accident and emergency area (staffed by frontline health workers including security, administrative clerks, porters, cleaners, nurses and medical doctors); iii) the outpatient department (with similar staff as the accident and emergency area); and iv) the SARS-CoV-2 dedicated wards for suspected and confirmed patients (staffed by specialized nursing, medical and cleaning staff). The assessment focused primarily on the “hierarchy of controls” areas of ventilation, administrative control, and personal protective equipment [30] for SARS-CoV-2. The two data collection tools were tested in an initial pilot with five hospitals from the participating PDoHs in April 2020, while the full PAR was conducted between April 28 and June 15, 2020.

### Variables measured

Variables of interest for the assessment of OSH readiness included the availability of a policy document to protect health workers, such as an OSH policy or IPC policy specific to the protection of health workers, the PDoHs coordination structure, and the availability of occupational medical doctor (OMP) or occupational health nurse (OHN). For the facility assessment, we calculated a total HealthWISE compliance score for each assessed area in the hospital, with a total possible score of 86 per hospital. The total HealthWISE scores were compiled from three categories: administrative control (possible score of 56), ventilation scores (possible score of 14), and PPE control (possible score of 16). We also collected hospital-specific information on infection rates among staff and the total number of employees.

### Data management and analysis

We performed double data entry and analysis on Microsoft Excel. We then imported the files into STATA version 16 (Stata Corp (2017) Stata Statistical Software: Release 15. College Station, TX: StataCorp LLC) for further analysis. The HealthWISE score out of 86 was calculated. The percentage compliance for each facility was calculated. Each health facility compliance score was graded according to Acceptable ≥75%; Requiring improvement (74–50%); and Unacceptable < 50%.

Logistic regression was used to model the association between HealthWISE scores (dependent variable) and the likelihood of a PDoH having a COVID-19 OHS policy or IPC policy (independent variable). We calculated the odds ratio and the respective 95% confidence intervals. Finally, we used Poisson regression to study the association between the number of cases at a hospital (dependent variable) and the compliance score (independent variable), offset by the total number of employees at the hospital.

### Intervention

This intervention was conducted in the context of health system strengthening, specifically with the goal of assisting the PDoHs and health facilities in protecting the health and safety of its limited health workers amidst the COVID-19 pandemic. Using PAR methods, the researchers provided a four-hour training on the data collection tools and the basics of the HealthWISE tool for each participating health facility and provincial department of health. This was followed by immediate data collection and administering of the adapted HealthWISE tool. Whenever the researchers identified gaps, the researchers and health facility personnel took immediate corrective advice and action. This action was followed by technical report briefings to provincial and hospital management on OSH and SARS-CoV-2 interventions for their province and health facility.

## Results

### Provincial assessment of OSH for health workers’ readiness for SARS-CoV-2

### Participant characteristics

Four PDoHs—representing 170,686 health workers—participated in the OSH readiness assessment. The OSH directorate represented provinces A and B. The Employee Health and Wellness directorate and the Department of Public Health Medicine represented Provinces C and D, respectively. By June 12, 2020, the four PDoHs had 3675 SARS-CoV-2 cumulative cases among health workers (2670 in Province A, 599 in Province B, 207 in Province C, and 199 in Province D).

### Provincial occupational health services readiness for the protection of health workers

At the time of data collection, all four provinces had recently developed provincial SARS-CoV-2 plan from which they were working, albeit not specific for OSH for health workers. In addition, provinces A and C had an IPC policy specific for SARS-CoV-2 in the health sector. Only Provinces A and D had OSH SARS-CoV-2 provincial coordinating teams and a dedicated budget for OSH.

At the PDoHs level, only Province A had an occupational medical practitioner. Provinces A and B had an occupational health nurse. All PDoHs had an IPC manager and an environmental health practitioner, except for Province C, which lacked the latter. Only Province A had an occupational hygienist, and only Province C lacked employee health and wellness professionals. Statutory laws in South Africa require employers to have health and safety representatives and committees, but only Province A and D had functional health and safety committees.

At the time of data collection, all PDoHs had provided SARS-CoV-2 training to a proportion of their health workforce. Province A provided the seasonal influenza vaccine to its health workers. Province D had conducted some health risk assessments specific to SARS-CoV-2. Provinces A and D reported having rehabilitation plans for infected and affected health workers and regularly screen them looking for incident cases. All Provinces but Province B provided treatment and mental health services. However, provinces B and C did not have adequate personal protective equipment.

All four PDoHs reported that they were collecting SARS-CoV-2 data from their health facilities using a tool (these differed in all the PDoHs). The PDoHs reasons for collecting data included reporting to National Government Authorities, including the Department of Health and Department of Employment and Labour, and the Department of Public Service and Administration, the employer of public servants in South Africa. Except for Province D, all the PDoHs indicated that they had a server to store the data, and none of the PDoH had a monitoring and evaluation plan.

### Health facility assessment based on principles of the HealthWISE tool

#### Health facilities and participants in the qualitative observational HealthWISE walkthrough risk assessment

Forty-five health facilities representing 34,192 health workers (i.e. 20% of the health workforce in the four PDoHs studied) participated and were trained in the qualitative observational HealthWISE walkthrough risk assessment for SARS-CoV-2 in the health facilities. The health workers who were trained and participated in the HealthWISE walkthrough risk assessment included: occupational medical practitioners, occupational health nurses, environmental health practitioners, IPC nurses, quality assurance nurses, and hospital managers (medical, nursing managers and finance). In addition, the sample included employee health and wellness practitioners and trade unions. Participation of occupational health doctors, occupational health nurses, EHPs, and IPC nurses depended on whether the hospital had such professionals on their staff establishment. As such, we used this indicator as a proxy for the availability of OSH services in that particular health facility.

#### HealthWISE walkthrough risk assessment findings for all facilities

The main entrance to the health facilities is the first contact between patients and the health facilities. It has a pedestrian gate or door-like structure and boom gate for vehicles which is manned by security guards. The walkthrough (Table 1) observed that the opportunity for close contact between security personnel and pedestrian patients as well as security personnel and drivers of vehicles was highly probable. Both the pedestrians and drivers had to sign in a security booklet; during this period, the opportunity of cross-infection increased due to the proximity of individuals, exchange of pens and booklets, and poor attention to IPC protocols.

**Table 1:** Overall main entrance HealthWISE walkthrough risk assessment findings

Main pedestrian and vehicle entrance to the health facility				
Prevention Measures	Question	% of facilities deemed adequate	% of facilities requiring improvement	Comments
**Ventilation**	Is outdoor ventilation adequate?	53	47	All outdoor entrances had good natural ventilation.
	Is indoor ventilation adequate?	0	100	No mechanical ventilation, windows & doors not open.
**Administrative measures**	Are patients social distancing?	54	46	Majority of staff not social distancing
	Are staff social distancing?	33	67	In the bigger facilities there were more security guards at the gate and there was lower likelihood of no social distancing.
	Are there markings for social distancing?	13	87	There were limited markings for social distancing
	Is furniture positioned for social distancing?	23	77	There was no rearrangement of furniture to allow for social distancing even when space allows
	Are there posters or information leaflets about COVID-19?	24	76	There were few COVID-19 posters in most of the facilities across the PDoH and when present did not address health workers but patients..
	Is there an area with water and soap to clean hands?	22	78	Lack of water basin and soap in certain areas particularly Province A was an issue but overall there was water and soap.
	Are there hand sanitizers in all entrances and exit points?	86	14	There seems to be huge investment in hand sanitizers
	Is waste properly segregated?	0	100	Majority of the facilities only had general waste bins and were not prepared for medical waste (SARS-CoV-2).
	Are COVID-19 waste management boxes	0	100	This was almost non-existent in the majority of facilities.
**Personal Protective Equipment**	Are workers wearing appropriate PPE?	18	82	At the entrance of the facility we expected surgical masks but majority of personnel were wearing cloth masks. There were also a few health workers who were wearing cloth mask inside the hospital. Since PPE is critical for this pandemic and investments have been huge, the facilities should train workers and supply appropriate PPE
	Are they wearing PPE correctly?	11	89	The surgical mask was sometimes below the nose and not covering both mouth and nose
	Are the staff wearing the same clothes from home, during work and back home?	0	100	Since may carry SARS-CoV-2 fomites on clothing, it is advisable that health workers change clothes to PPE on arrival and change back to their clothing when departing work.
	Do you think there is adequate PPE?	82	18	Generally, there was some form of PPE available

While ventilation in the main entrance was adequate for health workers in the outdoors security area, security guard houses were often small and crowded and lacked windows or any other form of ventilation. During the walkthrough, a lack of administrative control was immediately apparent, as there was a general lack of posters and signs. There were crowded chairs indicative of no social distancing, even in the absence of health workers in the area. The majority of security guards were wearing cloth masks but had them below their noses.

The accident and emergency areas in most health facilities were high-risk with a twenty-four-hour operation. We display the findings of the HealthWISE walkthrough risk assessment in Table 2. Ventilation was a considerable challenge in the majority of assessed health facilities. Natural ventilation was impaired by mostly poor facility design, while mechanical ventilation was not available in 82% of facilities and when available was broken and had a poor maintenance record. In the health facilities assessed early on in the study, we noticed that administrative controls would only be followed and monitored during working hours. Afterhours, there is no extra staff assisting with the enforcement of IPC measures, leading to a lack of implementation and monitoring of IPC measures for evening and night shifts.

**Table 2:** Overall Accident and Emergency area HealthWISE walkthrough risk assessment findings

Accident and Emergency Department				
Prevention Measures	Question	% of facilities deemed adequate	% of facilities requiring improvement	Comments
**Ventilation**	Is natural ventilation adequate?	25	75	Poor culture of opening windows,
	Is mechanical ventilation adequate	18	82	lack of mechanical ventilation
**Administrative measures**	Are patients social distancing?	58	42	Most of the facilities have provisions for patients social distancing such as markings and queue marshals.
	Is staff social distancing?	29	71	Majority of the staff were not practicing social distancing particularly when they were in their workstations, there is a space shortage
	Are there markings for social distancing?	51	49	Inside the area there was a considerable number of institutions without markings for social distancing in their accident and emergency area.
	Is furniture positioned for social distancing?	25	75	There was no rearrangement of furniture to allow for social distancing mostly due to lack of space.
	Are the posters or information leaflets about COVID-19?	25	75	Majority of the facilities have few and poor quality Covid-19 posters and very few facilities have posters that addresses OSH of HWs against Covid-19
	Is there an area with water and soap to clean hands?	97	3	With regards to the few facilities that did not have water, it was a municipality issue of a water outage in the whole municipality during the walk through
	Are there hand sanitizers in all entrances and exit points?	58	42	Some accident and emergency departments did not have visible hand sanitizers in their entrances or exits. One of the challenges mentioned was to put them in the strategic positions e.g. entrances and exit points
	Is waste properly segregated?	92	8	
	Are COVID-19 waste management boxes?	72	28	
**Personal Protective Equipment**	Are workers wearing appropriate PPE?	89	11	There were those few health workers that were not wearing appropriate PPEs e.g. cloth masks in accident and emergency
	Are workers wearing PPE correctly?	36	64	The surgical mask were sometimes below the nose
	Are the staff wearing the same clothes from home, during work and back home?	0	100	Since may carry SARS-CoV-2 fomites on clothing, it is advisable that health workers change clothes to PPE on arrival and change back to their clothing when departing work.
	Do you think there was adequate PPE?	94	6	Generally, there was some form of PPE available

In the accident and emergency department, the social distancing of staff was a great challenge due to insufficient space and high patient load. In some of the health facilities assessed, we found that they had or planned to have makeshift working areas using tents in some instances. This arrangement led to overcrowding, more pronounced in urban facilities. Health facilities in rural areas had high patient volumes in the mornings and almost no patients in the afternoons, allowing for social distancing. Health workers across facilities were often found congregating when engaging in administrative work or not attending to patients. There were many facilities with small or no rest areas leading to health workers crowding the administrative areas for their paperwork and resting periods. It was, however, concerning that there were only 25% of health facilities with SARS-CoV-2 posters or educational materials. Waste management was generally good in the majority of assessed health facilities. Eighty-nine (89) percent of the health workers were wearing the appropriate PPE, but only 36% of health facilities were wearing it correctly. In all the health facilities, health workers in the accident and emergency department would come to work in their uniform, work with it and return home wearing it, though in most cases the health workers would wear a disposable over gown with their clothing.

The findings for the outpatient departments in the assessed health facilities (Table 3) were similar to the accident and emergency department walkthrough. About 20% of health facilities assessed had natural ventilation and only 4% had mechanical ventilation, with Province A facilities being most affected by poor ventilation. Social distancing was adhered to and deemed adequate in 67% of health facilities by their patients. Most health facilities aided this by marking brightly and visibly cancelling out some chairs or seating areas and rendering them unavailable for use. Patients standing in queues were assisted with markings of distances of between ≥1.5 m to maintain social distance. Health workers had no or low access to often small dining and rest areas, leading to poor social distancing. Several health facilities had rearranged the furniture to allow for social distancing, albeit not adequately due to poor infrastructure and lack of space. A notable issue was the lack of posters and information on how health workers can protect themselves from being infected with SARS-CoV-2. There was access to water, soap and sanitizers for hand hygiene and proper management of waste from SARS-CoV-2 contaminated materials. On observation, workers were wearing PPE though it varied with a few wearing cloth masks and surgical masks and many using respirators, particularly N95 respirators.

**Table 3:** Overall Outpatient Department HealthWISE walkthrough risk assessment findings

Outpatient Department				
Prevention Measures	Question	% of facilities deemed adequate	% of facilities requiring improvement	Comments
**Ventilation**	Is natural ventilation adequate?	20	80	The majority of the Province A facilities required a lot of improvement, there was poor natural ventilation
	Is there adequate mechanical ventilation	4	96	There was no mechanical ventilation, on in few facilities
**Administrative measures**	Are patients social distancing?	67	33	Very few facilities were not ensuring that patients are observing social distancing. A few health facilities were using queue marshals to control social distancing.
	Are staff social distancing?	25	75	Health workers concentrated around administrative desks to write notes, use telephone, complete forms, and even socialize. Small or lack of dining and rest areas contributed.
	Are there markings for social distancing?	49	51	These were lacking in most OPDs
	Is furniture positioned for social distancing?	40	60	Some hospitals had not rearranged the furniture for social distancing as the distance between the furniture was the same as before the Covid-19 pandemic, but some did not have space to change arrangement
	Are the posters or information leaflets about COVID-19?	16	84	A few of the facilities had COVID-19 posters but they did not indicate how health workers can protect themselves and they were of poor quality
	Is there an area with water and soap to clean hands?	96	4	With regards to the few facilities that did not have water, it was a municipality issue of a water outage in the whole municipality during the walk through
	Are there hand sanitizers in all entrances and exit points?	58	42	
	Is waste properly segregated?	93	7	
	Are COVID-19 waste management boxes present	74	26	
**Personal Protective Equipment**	Are workers wearing appropriate PPE?	93	7	
	Are they wearing PPE correctly?	29	71	The surgical mask was sometimes below the nose
	Are the staff wearing the same clothes from home, during work and back home?	0	100	Since may carry SARS-CoV-2 fomites on clothing, it is advisable that health workers change clothes to PPE on arrival and change back to their clothing when departing work.
	Do you think there was adequate PPE	97	3	Generally there was some form of PPE available

The wards dedicated to suspected and confirmed SARS-CoV-2 patients varied considerably within and between provinces (Table 4). Some facilities did not have these wards or were still in the preparatory stages for these wards (i.e. renovations were taking place). Ventilation in these wards was mechanized and working in the facilities with dedicated wards for SARS-CoV-2 patients. However, in Province A, there were a few health facilities with poor ventilation with non-functioning mechanical ventilation. In almost all health facilities assessed, the suspected and confirmed SARS-CoV-2 patients were socially distancing or kept in physically separated rooms. Health workers were social distancing in the main, except in a few health facilities in Province A, where we found health workers seated close to the main administration desk area when not attending to patients. Most of the health facilities had set up the furniture to allow for social distancing.

**Table 4:** Overall SARS-CoV-2 dedicated wards for suspected and confirmed patients HealthWISE walkthrough risk assessment findings

SARS-CoV-2 dedicated wards for suspected and confirmed patients				
Prevention Measures	Question	% of facilities deemed adequate	% of facilities requiring improvement	Comments
**Ventilation**	Is natural ventilation adequate?	57	43	Some of the Province A facilities need significant improvements
	Is there adequate mechanical ventilation	21	79	
**Administrative measures**	Are patients social distancing?	83	17	All of the patients under investigation were practicing social distancing
	Is staff social distancing?	83	17	Particularly during rest periods and when doing administrative work
	Is furniture positioned for social distancing?	81	19	
	Are there posters or information leaflets about COVID-19?	11	89	Few of the facilities had COVID-19 posters but they did not indicate how health workers can protect themselves and they were of poor quality.
	Is there an area with water and soap to clean hands?	97	3	During the walkthrough, one facility in Province B did not have water as there was water outage in the whole town.
	Are there hand sanitizers at all entrances and exit points?	95	5	
	Is waste properly segregated?	94	6	
	Are COVID-19 waste management boxes present	79	21	
**Personal Protective Equipment**	Are workers wearing appropriate PPE?	85	15	
	Are they wearing PPE correctly?	86	14	
	Is the staff wearing same clothes from home, during work and back home?	5	95	Since may carry SARS-CoV-2 fomites on clothing, it is advisable that health workers change clothes to PPE on arrival and change back to their clothing when departing work.
	Do you think there is adequate PPE	70	30	

The posters in these wards were not specific for OSH or the protection of health workers, and their placement in the wards was not ideal for easy access by health workers. The facilities had water, soap and sanitizers except for one health facility with no water due to municipality failures for the whole town. Sanitizers, while present, were not strategically placed for easy access by health workers. Waste management was adequate, and health workers were able to discard with ease all contaminated materials. Health workers had access and were wearing PPE correctly with the exception of a few health facilities in Province A. Some health facilities in Province C displayed some elements of best practice as they had a suspect and confirmed SARS-CoV-2 patient ward separate from the rest of the hospital building, and the health workers for that ward were isolated from the rest of the hospital, stayed in accommodation provided by the hospital, and utilized hospital clothing and PPE during their shifts.

Table 5 presents the individual HealthWISE scores per health facility. The lowest was 14/86, while the highest was 64/86 (median 40, IQR 25–52), administrative scores were from 6/56–46/56 (median 28, IQR 17–36), ventilation scores were from 0/14–11/14 (median 4, IQR 2–6), and PPE scores were from 3/16–15/16 (median 9, IQR 7–11). None of the health facilities met the criteria for acceptable HealthWISE score of ≥ 75%, and 42% had an unacceptable HealthWISE score of below 50%.

**Table 5:** HealthWISE compliance scores

Province	Hospital	Ventilation score (14)	Administrative score (56)	PPE score (16)	Total Score (86)	Mean Score	Acceptable compliance (≥ 75%)
Province A	PA 1	11	24	10	45	15	No = 52,3%
	PA 2	0	16	6	22	7	No = 25,6%
	PA 3	4	41	11	56	19	No = 65,1%
	PA 4	0	25	9	34	11	No = 39,5%
	PA 5	0	35	10	45	15	No = 52,3%
	PA 6	0	16	6	22	7	No = 25,6%
	PA 7	2	8	10	20	7	No = 23,3%
	PA 8	0	11	9	20	7	No = 23,3%
	PA 9	2	6	10	18	6	No = 20,9%
Province B	PB 1	4	37	8	49	16	No = 57,0%
	PB 2	9	34	8	51	17	No = 59,0%
	PB 3	5	37	11	53	18	No = 62,0%
	PB 4	3	28	7	38	13	No = 59,0%
	PB 5	4	26	8	38	13	No = 59,0%
	PB 6	11	43	10	64	21	No = 74,0%
	PB 7	10	36	10	56	19	No = 66,0%
	PB 8	8	37	10	55	18	No = 64,0%
	PB 9	7	36	9	52	17	No = 60,0%
	PB 10	6	35	9	50	17	No = 58,0%
	PB 11	5	10	7	22	7	No = 34,0%
Province C	PC 1	7	40	15	62	20	No = 72,0%
	PC 2	6	42	15	63	21	No = 73,0%
	PC 3	6	33	15	54	18	No = 63,0%
	PC 4	6	30	15	51	17	No = 59,0%
	PC 5	6	46	11	63	21	No = 73,0%
	PC 6	5	23	11	39	13	No = 45,0%
	PC 7	5	36	15	56	19	No = 65,0%
	PC 8	3	32	11	46	15	No = 53,0%
	PC 9	5	42	11	58	19	No = 67,0%
	PC 10	8	42	12	62	20	No = 72,0%
Province D	PD 1	5	17	7	29	10	No = 34,0%
	PD 2	4	24	6	34	11	No = 53,0%
	PD 3	7	20	9	36	12	No = 42,0%
	PD 4	2	12	4	18	6	No = 43,0%
	PD 5	4	21	7	32	11	No = 50,0%
	PD 6	6	31	5	42	14	No = 49,0%
	PD 7	3	12	7	22	7	No = 34,0%
	PD 8	6	28	10	44	15	No = 51,0%
	PD 9	4	23	9	36	12	No = 42,0%
	PD 10	2	16	3	21	7	No = 50,0%
	PD 11	2	10	4	16	5	No = 38,0%
	PD 12	2	19	4	25	8	No = 60,0%
	PD 13	3	28	10	41	14	No = 47,0%
	PD 14	4	31	10	45	15	No = 52,0%
	PD 15	1	9	4	14	5	No = 33,0%

The results of the logistic regression analysis assessing the availability of a provincial policy and hospital preparedness indicators showed that PPE and ventilation scores were statistically associated with the availability of a COVID-19 provincial policy (Table 6). The presence or implementation of the provincial policy was associated with an increase in PPE score.

**Table 6:** Logistic Regression model assessing the association between the Provincial Policy and indicators for hospital preparedness

Variables	Odds Ratio (95% CI)	***P*** Value
PPE Score	2.58 (1.66–4.91)	< 0.001
Ventilation Score	0.57 (0.34–0.82)	0.011

#### Hospital staff infection rate and compliance score

**Fig. 1:**
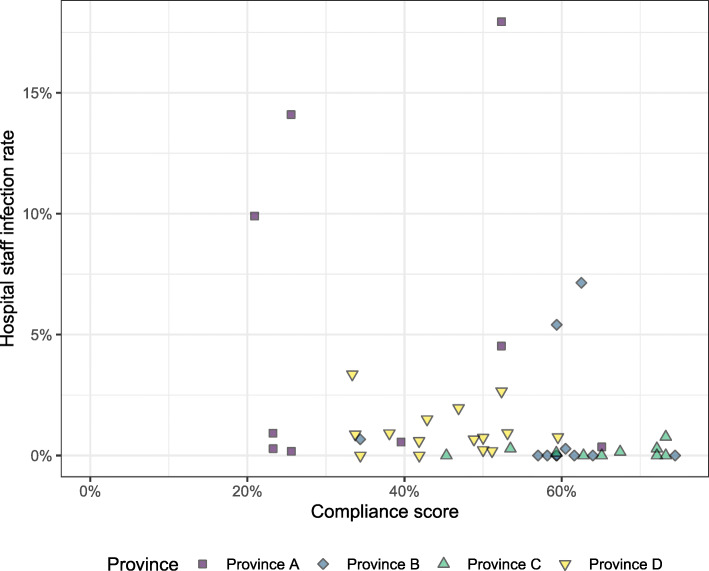
Infection rate by the compliance score of each hospital, indicated by province

From March 5, 2020 to June 15, 2020, the cumulative hospital infection rate for COVID-19 at the 45 facilities ranged from 0 to 17.9%, with a median 0.3% infection rate (IQR: 0.0–0.9%). We applied nonparametric statistical tests because the data seemed non-normal (Shapiro-Wilk test: *p* < 0.001). We further adjusted by the total number of employees given that plotting infection rate against compliance score showed there might be some clustering by province (Fig. 1).

**Fig. 2:**
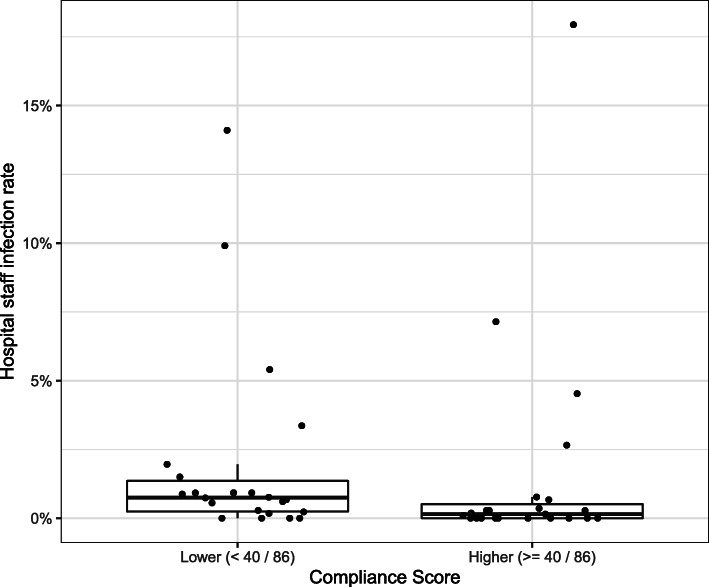
Box plot comparing lower and higher compliance hospital infection rates. Each point represents a hospital. The boxes show the IQR (25th to 75th percentile) with the bold horizontal line through the centre of the box showing the median

We classified each hospital into two categories: lower compliance or higher compliance, using the median score (40) to determine categories (Fig. 2). The lower compliance group (with a score < 40) had a median infection rate of 0.75%, and the higher compliance group (with score ≥ 40) had a median infection rate of 0.15%. While the infection rate is low for both groups, the Mann-Whitney U test showed a significant difference between the medians of the two groups (*p* = 0.03). Our adjusted Poisson Regression estimated an incidence rate ratio of 0.89 (95% Confidence Interval: 0.86, 0.91) for each 5% increment in the compliance score.

## Discussion

When we conducted our study, the SARS-CoV-2 epidemic was still moving towards its peak with at least 106,108 cases and the epidemic curve still rising sharply in South Africa [31, 32]. The national data indicated SARS-CoV-2 cases clustering among health workers in some health facilities, a phenomenon that was expected with plans to contain it.

Our PAR conducted early in the pandemic aimed to assess the OSH system for health worker’s readiness for SARS-CoV-2; and to assess health facilities for the protection of health workers against SARS-CoV-2 infection with reference to the principles of the HealthWISE tool. The PAR methodology allowed for all participating health worker irrespective of level of education or job category to actively participate and be a ‘change agents’. This, while getting empowered to manage SARS-CoV-2 in their own workplace. Participating health workers, utilizing PAR and HealthWISE tool principles were able to willingly identify SARS-CoV-2 IPC and OSH challenges; contextually appropriate, local and acceptable solutions as well as on an ongoing basis with the researcher playing a facilitator and creating opportunities for change [33].

### Provincial departments of health OSH readiness

There was a clear discrepancy in the availability of resources for OSH systems and their readiness for SARS-Cov-2 in the four PDoH. The Province A, which is in the economic hub of South Africa, was more prepared and had more resources compared to the other three PDoHs, which are mostly rural. While only Province A and D already had a policy on SARS-CoV-2 in healthcare, all the PDoH reported having health plans for protecting health workers from SARS-CoV-2. This finding is consistent with studies examining TB IPC for health workers where it was found that South Africa had IPC policies but lacks in implementation [34, 35]. It was not surprising that Province A, had an allocated budget for the protection of health workers from SARS-CoV-2, as it generally has more resources than other PDoH in South Africa [36].

However, it was unexpected for Province D, since it is generally a poorly resourced PDoH with limited human resources for OSH, particularly occupational medical and nursing professionals. While of serious concern, it was not surprising that only Province A had an occupational medical practitioner driving the programme at the provincial level. Province B had occupational health nursing support at a provincial level, which is common in South Africa, hence it was troubling that the Province C had no medical or nursing support. This lack of occupational health trained personnel at provincial level to assist in planning and managing the OSH system for health workers in the fight against SARS-CoV-2 suggested to the authors that major gaps may exist in the protection of health workers. While IPC nurses were present in the health system, they did not play an active role at a provincial or facility level in protecting the health of health workers, their role was around general IPC for the patients in main. It was however, a more pressing emergency in Province C that they did not have any of the essential human resources for the fight against SARS-CoV-2 and or even OSH in general. This shortage of OSH professionals not only in Province C but also in Province B and D may constitute an obstacle to the provision of OSH services [28].

Since our PAR was conducted in the early stages of the epidemic, we hoped to check if the PDoHs were prioritizing primary prevention and we were disappointed to learn that only one PDoH had carried out the health risk assessments and two had procured PPE. While the South African National Department of Health had issued a directive for health workers to receive the influenza vaccine, only Province D had started offering its health workers influenza vaccinations. All the PDoH reported providing education and training, albeit on general SARS-CoV-2 information. There was some information related to the public health and IPC response that was similar for OSH. It was impressive that three of the PDoH were providing screening and testing, treatment and mental health services for their health workers.

Overall, Province A, B and D had some elements of an OSH system that were activated to protect the health workers. While our PAR was targeted at assessing the OSH system readiness for SARS-CoV-2 in PDoHs, it has been able to identify the coverage of OSH for health workers in line with calls from numerous sources including the United Nations Resolution on Sustainable Development Goals [37, 38]. As shown by Rantanen et al. [28], our study identified that all the assessed PDoHs had some OSH systems coverage albeit of varying coverage and resources between the PDoHs, with Province A being the most resourced and Province C the least resourced. Our study further confirms the importance of having a health policy in place for health interventions, as shown by the fact that the odds of having an acceptable PPE score were 72% if the PDoH had a SARS-CoV-2 policy for health workers.

### HealthWISE walkthrough risk assessment

Our research team has previously trained health workers in South Africa, particularly in Province A and B on the use of HealthWISE [21, 22]; Using an adapted HealthWISE tool health workers and health facility managers identified SARS-CoV-2 IPC measures. Based on the observation of our research team using the HealthWISE tool, our research showed that in South Africa, OSH for health workers was occupational health nurse driven and with a large number of environmental health practitioners supporting at health facilities, with a significantly low number of occupational medical practitioners. It was concerning for the research team to find that only about half of assessed health facilities had IPC nurses. We were encouraged by the participation of very senior health facility management including chief executives, medical managers and nursing matrons highlighting how seriously some health facilities were taking the SARS-CoV-2 epidemic; as well as their understanding and embracing of the HealthWISE way of doing OSH.

Our HealthWISE adapted tool concentrated in some key areas of the health facilities to provide a snapshot of what health workers and managers needed to prioritize using limited resources. The research identified the health facility entrance as the main area of strategic importance for not only alerting and increasing health workers’ OSH practice, but also for influencing their change of behavior under the new normal of SARS-CoV-2. Overall, the HealthWISE tool indicated that health facilities had not identified and actively prepared their health facilities to protect health workers, patients and visitors from SARS-CoV-2. The fact that none of the assessed health facilities attained the acceptable HealthWISE score of ≥75% was concerning, highlighting the need for increased effort to protect the health of health workers. This poor performance also highlighted the importance of a pragmatic tool, such as HealthWISE, which can be utilized by all health workers in order to monitor and evaluate the performance of their OSH in their workplace without the need for highly specialized professionals. During the HealthWISE risk assessments, we encountered a wide range of IPC and OSH practices from non-existent to excessive measures between and within the PDoHs, even though South Africa has produced national IPC guidelines for health settings [39]. Finally, our analysis of the relationship between OSH compliance and infection rates in health workers importantly showed that those facilities with a score above the median had a reduced risk compared to those below the median, which suggests that facilities should aim to improve their score even when small improvements do not lead to an acceptable level of compliance.

Overall, we found that a health policy is essential for the implementation of an OSH programme for health workers and that the HealthWISE approach to assessing health facilities’ IPC and OSH readiness for SARS-CoV-2, was very useful for health workers and management without formal training in both IPC and OSH. It also allowed for a more open, non-threatening and acceptable way of bringing about change as the managers and health workers were able to identify gaps and recommend feasible interventions often at no cost or within reasonable cost to both the PDoH and health facility.

### Limitations

As our study was conducted as part of a rapid appraisal of COVID-19 OSH preparedness early in the pandemic in South Africa, the need to provide OSH services at the earliest possible time to protect the limited health workforce necessitated the use of operational research methods. Hence the use of PAR, which may affect the validity of the research since the researchers are also part of the implementation, though this approach did allow for strengthened implementation. We used purposive sampling to select the four PDoHs OSH managers from whom we sought responses on the SARS-CoV-2 readiness of the provincial governments; however, the possibility of “socially desirable answers” cannot be excluded. Our data collection instruments for both SARS-CoV-2 readiness and HealthWISE were specifically designed for this study and the nature of the research under the COVID-19 pandemic being a rapid appraisal under limited time may affect the validity of our research. Our research gives a good indication of the state of OSH, particularly COVID-19 readiness of the health facilities included in our study but the non-probability sampling strategy and potential biases in qualitative assessments may limit the generalization of the findings.

While, this paper reports on assessing the readiness of OSH systems in place to protect health workers from SARS-CoV-2 and conducting a health facility assessment for the protection of health workers against SARS-CoV-2 infection based on principles of the HealthWISE tool. It is part of a larger study which also interviewed 45 respondents [40] based at hospital level and also interviewed 286 frontline health workers [26], who helped triangulate and interpret the overall results, and assisted with validation of the truthfulness of the responses provided by the OSH managers. Importantly, as noted above, respondents included trade unionists and personnel at other levels within the healthcare system who did not have any incentive to provide an unwarranted favourable perspective.

## Conclusions

While South Africa is an upper-middle-income country, there are vast differences in the resources for health between and within PDoH. Our study was able to show these differences, with one province proving to have abundant OSH resources, two provinces having some resources, while one province was severely under-resourced in terms of IPC and OSH. Our timely assessment of the OSH system for SARS-CoV-2 also highlighted the variability in coverage and services between and within PDoH, and allowed for timely alerts to the relevant authorities so that appropriate interventional steps could be undertaken. At a health facility level, the role of managers and health workers collaborating in improving the IPC and OSH measures using the HealthWISE approach proved to be an acceptable and appreciated method. It would be valuable to assess how regular application of such self-assessment methods can and do stimulate improvement in the processes of providing protection and in producing improved results as the COVID-19 and other health risks endure.

## Supplementary Information

**Additional file 1: Appendix 1**. How the semi-structured HealthWISE walk-through risk assessment survey was conducted.

## Data Availability

The datasets used and analyzed during the current study are available from the corresponding author on request.

## Abbreviations

COVID-19: Coronavirus disease 19
OSH: Occupational safety and health
SARS-CoV-2: Severe acute respiratory syndrome coronavirus 2
WHO: World Health Organization
