# Supplementary material for: Organizational factors associated with health worker protection during the COVID-19 pandemic in four provinces of South Africa
Source: BMC Health Serv Res. 2021 Oct 11;21:1080. doi: 10.1186/s12913-021-07077-w (PMC8504782; doi:10.1186/s12913-021-07077-w)
Supplement: Supplementary file 1 — Additional file 1: Appendix 1. How the semi-structured HealthWISE walk-through risk assessment survey was conducted. [file 12913_2021_7077_MOESM1_ESM.pdf]

Appendix 1

How the semi-structured HealthWISE walk-through risk assessment survey was conducted:

1. This section is driven by the HealthWISE Tool of the World Health Organization (WHO) and the International Labour Organization (ILO). Health workers (HWs) in the hospitals have been trained on a basic methodology for conducting a walk-through survey in the areas where they work and observe the working environment for biological hazards. This is then followed by a short working discussion on what they can do to improve the working environment without engaging OHS professionals or even external experts.

2. The procedure for this walk-through is as follows:

   i. Walkthrough team (Researchers, provincial OHS coordinator, health facility OHS coordinator, and one person from the area (e.g. casualty) being assessed);

   ii. Before entering the area, the team is offered surgical masks ONLY as personal protective equipment (PPE), and they use hand sanitizer on entry of the area walk-through survey;

   iii. The four walk across the area (maintaining social distancing) and look & observe without any discussion while in the area (casualty);

   iv. On exit the group again uses the hand sanitizers to clean their hands;

   v. The group then finds an open area without activity to then have a discussion on what they have observed and what immediate recommendation, and what might require further discussion with health facility management that the hospital OHS coordinator will take to management;

      1.

   vi. At the discussion each person writes their own notes for discussion with the hospital OHS coordinator expected to implement the discussion working with the ward managers and hospital management as per the guidelines of the principles of the WHO/ILO HealthWISE Tool.

vii. It must be noted that the researcher / academic support is not going to be available for all the hospitals and the hospital OHS coordinator is expected to learn from the walk-through so they can escalate the service to other health facilities within their PDoH;

Areas to be visited

<u>Entrance gates (pedestrian and cars)</u>

1. Ventilation

   Is it adequate OR not adequate? Elaborate below

2. Administrative controls
   a. Are patients social distancing?

   b. Is staff social distancing?

   c. Are there markings for social distancing?

   d. Is furniture positioned for social distancing?

   e. Are the posters or information leaflets about COVID-19?

   f. Is there an area with water and soap to clean hands?

g. Are there hand sanitizers in all entrances and exit points?

h. Is waste properly segregated?

i. Are COVID-19 waste management boxes or even leaflets

j. Any other observation

3. Personal Protective equipment (PPE)
   a. What PPE are staff in this area wearing?

   b. Are they wearing PPE correctly

   c. Is staff wearing their home clothes

   d. Do you think there is adequate supply of PPE

<u>Corridors and passages</u>

1. Ventilation

   Is it adequate OR not adequate? Elaborate below

2. Administrative controls
   a. Are patients social distancing?

b. Is staff social distancing?

c. Are there markings for social distancing?

d. Is furniture positioned for social distancing?

e. Are the posters or information leaflets about COVID-19?

f. Is there an area with water and soap to clean hands?

g. Are there hand sanitizers in all entrances and exit points?

h. Is waste properly segregated?

i. Are COVID-19 waste management boxes or even leaflets

j. Any other observation

3. Personal Protective equipment (PPE)
    a. What PPE are staff in this area wearing?

b. Are they wearing PPE correctly

c. Is staff wearing their home clothes

d. Do you think there is adequate supply of PPE

Casualty

1. Ventilation

   Is it adequate OR not adequate? Elaborate below

2. Administrative controls
   a. Are patients social distancing?

   b. Is staff social distancing?

   c. Are there markings for social distancing?

   d. Is furniture positioned for social distancing?

   e. Are the posters or information leaflets about COVID-19?

   f. Is there an area with water and soap to clean hands?

g. Are there hand sanitizers in all entrances and exit points?

h. Is waste properly segregated?

i. Are COVID-19 waste management boxes or even leaflets

j. Any other observation

3. Personal Protective equipment (PPE)
    a. What PPE are staff in this area wearing?

    b. Are they wearing PPE correctly

    c. Is staff wearing their home clothes

    d. Do you think there is adequate supply of PPE

OPD

1. Ventilation

   Is it adequate OR not adequate? Elaborate below

2. Administrative controls
    a. Are patients social distancing?

b. Is staff social distancing?

c. Are there markings for social distancing?

d. Is furniture positioned for social distancing?

e. Are the posters or information leaflets about COVID-19?

f. Is there an area with water and soap to clean hands?

g. Are there hand sanitizers in all entrances and exit points?

h. Is waste properly segregated?

i. Are COVID-19 waste management boxes or even leaflets

j. Any other observation

3. Personal Protective equipment (PPE)
   a. What PPE are staff in this area wearing?

b. Are they wearing PPE correctly

c. Is staff wearing their home clothes

d. Do you think there is adequate supply of PPE

<u>COVID-19 ward (isolation)</u>

1. Ventilation

   Is it adequate OR not adequate? Elaborate below

2. Administrative controls
   a. Are patients social distancing?

   b. Is staff social distancing?

   c. Are there markings for social distancing?

   d. Is furniture positioned for social distancing?

   e. Are the posters or information leaflets about COVID-19?

   f. Is there an area with water and soap to clean hands?

g. Are there hand sanitizers in all entrances and exit points?

h. Is waste properly segregated?

i. Are COVID-19 waste management boxes or even leaflets

j. Any other observation

3. Personal Protective equipment (PPE)
    a. What PPE are staff in this area wearing?

    b. Are they wearing PPE correctly

    c. Is staff wearing their home clothes

    d. Do you think there is adequate supply of PPE
